# Vector Atomic Magnetometer with Free Induction Decay Detection Based on a Microfabricated Vapor Cell

**DOI:** 10.3390/mi16010041

**Published:** 2024-12-30

**Authors:** Pengbo Jiang, Qi Li, Jianan Qin, Zhiyuan Jiang

**Affiliations:** 1 Center for Advanced Measurement Science, National Institute of Metrology, Beijing 100029, China; jiangpb@nim.ac.cn (P.J.); jiangzy@nim.ac.cn (Z.J.); 2 School of Optical and Electronic Technology, China Jiliang University, Hangzhou 310018, China

**Keywords:** vector atomic magnetometer, free induction decay, MEMS vapor cell

## Abstract

Atomic magnetometers are highly sensitive instruments widely used for measurements of weak magnetic field. Extracting vector information while maintaining high-precision scalar detection has become the trend in atomic magnetometer development. We introduce a vector atomic magnetometer containing a 5 mm-thick microfabricated vapor cell operating in free-induction-decay mode. By employing orthogonal modulation techniques, the system achieves high-precision in-plane vector magnetic field measurements. The high-precision vector magnetic field measurements are demonstrated in the *x–z* plane. The sensitivity of the total field detection in the miniaturized atomic magnetometer is 30 pT·Hz^−1/2^ @11 µT. The average angular error of the decoupled measurement is as low as 4.7 mrad @7.6 µT for vector magnetic fields, providing a new approach for vector magnetic field measurement in miniaturized atomic magnetometers.

## 1. Introduction

Atomic magnetometers exhibit high sensitivity in weak magnetic field measurements and have found wide applications in fields such as geophysical exploration [1,2,3,4,5,6,7,8], geomagnetic navigation [9], and biomedical research [10]. Advances in miniaturization technologies and integrated optics have progressively reduced the size of atomic magnetometers. These miniaturized designs not only significantly lower system complexity but also enhance portability and adaptability to various environmental conditions [11]. As the demand for comprehensive magnetic field information increases, scalar magnetometers, which only measure magnetic field strength, are insufficient for resolving spatial magnetic field distributions. In this context, vector magnetometers have increasingly gained importance. For example, vector information significantly improves positioning accuracy in geomagnetic navigation and provides more comprehensive data for studying neural activities, such as in magnetoencephalography (MEG) [12,13]. Miniaturized vector magnetometers combine portability with vector detection capabilities, facilitating their use in diverse and complex environments such as embedded navigation systems, space exploration probes, and wearable medical devices [14]. This integration of vector detection and miniaturization not only broadens their application scenarios but also enables multisensor integration, driving the advancement of precision magnetic field measurement technologies toward greater portability and multifunctionality.

In recent years, significant progress has been made in the development and optimization of miniaturized vector magnetometers. B. Patton utilized the nonlinear magneto-optical rotation effect of atoms to measure magnetic field strength and direction. By applying laser AC Stark shifts along cross axes to generate equivalent cross magnetic fields, the magnetic resonance frequency was demodulated to extract the field components. This method achieved a root mean square (RMS) noise floor of approximately 65 pT·Hz^−1/2^ for magnetic field strength measurements and 0.5 mrad·Hz^−1/2^ for directional measurements [15]. The vector atomic magnetometer, leveraging this approach, demonstrates exceptional sensitivity, an all-optical design, and robust interference suppression, making it highly advantageous for precision magnetic field measurements and vector field detection. However, the stringent requirements for optical shift beam stability, the complexity of the system architecture, and the need for external magnetic shielding impose limitations on its applicability in portable and rapid dynamic magnetic field measurements. Xin Meng combined machine learning with atomic magnetometers by using a pretrained deep neural network to map recorded signals to vector field information, constructing a fully optical vector atomic magnetometer. This system demonstrated a sensitivity of approximately 100 fT·Hz^−1/2^ for magnetic field strength measurements and 100∼200 µrad·Hz^−1/2^ for angular measurements [16]. The magnetometer leverages machine learning to simplify sensor architecture, enabling miniaturization and compact design, having the potential for real-time dynamic magnetic field measurements, but its performance is constrained by electronic noise, signal angle degeneracy, and resonance linewidth limitations. Building upon the spin-exchange relaxation-free (SERF) atomic magnetometer, Zhang JiaLong configured a system as a triaxial vector magnetometer by implementing a coordinate system rotation. By rotating the original coordinate system by 45°, the system’s response was enhanced along the nonsensitive axis and reduced along the sensitive axis. After the rotation, the sensitivities along the *x*, *y*, and *z* axes were measured to be 55 fT·Hz^−1/2^, 38 fT·Hz^−1/2^, and 60 fT·Hz^−1/2^, respectively [17]. This design improves sensitivity uniformity across three axes and facilitates triaxial vector measurement; however, the system’s performance is limited by weakened z-axis signals under low magnetic fields, reduced sensitive axis performance due to coordinate offset, and interaxis coupling effects that require further investigation. These miniaturized vector magnetometer designs primarily rely on vapor cells fabricated using glassblowing techniques [18,19]. However, glass-blown vapor cells face challenges such as uneven heating caused by residual glass tails, which hinder further miniaturization and integration. Moreover, glassblowing is a low-efficiency process that is difficult to scale for mass production. MEMSs (micro-electromechanical systems) technology has emerged as an ideal alternative to address these limitations [20,21]. The MEMS vapor cell is free from tail structures, significantly improving heating uniformity and facilitating further miniaturization and integration. Additionally, the batch production capability of MEMS processes ensures high-quality vapor cells with greater efficiency and reduced costs, meeting the demands of large-scale applications. To be best of our knowledge, the current literature indicates that research on miniaturized vector atomic magnetometers based on MEMS vapor cells remains relatively limited. Therefore, our research focused on the integration of MEMS vapor cells with miniaturized vector atomic magnetometers. By incorporating vapor cells fabricated using MEMS technology into the miniaturized atomic magnetometer, we aimed to develop a compact vector magnetometer system. Building upon this foundation, modulation techniques were introduced to configure the miniaturized atomic magnetometer for vector detection, ultimately producing a miniaturized vector atomic magnetometer system based on MEMS vapor cells.

In this paper, we propose a miniaturized free induction decay (FID)-based vector atomic magnetometer utilizing a microfabricated thick MEMS vapor cell. This design enables a single-beam, vector magnetic field measurement. A rubidium vapor cell with a diameter of 4 mm and a thickness of 5 mm was fabricated using MEMS technology, and a compact FID-based magnetometer probe was designed. Total magnetic field detection was achieved by extracting and fitting FID signals. Modulation fields were applied along two axes perpendicular to the pump beam, enabling the decoupling of magnetic field components along these axes and extracting vector magnetic field measurements within the *x–z* plane perpendicular to the pump beam. This method eliminates the need for external RF driving to sustain resonance, effectively avoiding additional external interference.

## 2. Materials and Methods

**Figure 1:**
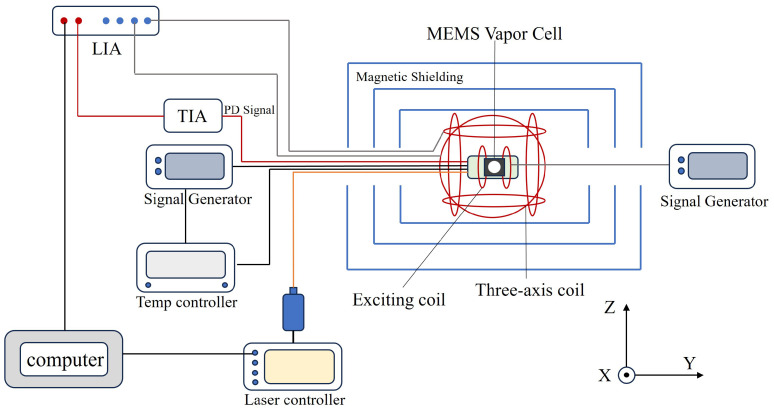
Schematic of the FID-based atomic magnetometer system.

The free induction decay (FID)-based atomic magnetometer system was constructed in this study, as shown in Figure 1. The system consisted of a magnetic shielding barrel, a miniaturized atomic magnetometer sensor, excitation coils, a triaxial Helmholtz coil, a signal generator, a temperature controller, a transimpedance amplifier, a lock-in amplifier, and a tunable 795 nm semiconductor laser. Since the MEMS vapor cell must be heated to high temperatures to achieve a high atomic density, the sensor housing must be made of high-temperature-resistant and deformation-resistant materials. Therefore, the housing of the miniaturized atomic magnetometer sensor was fabricated from polyether ether ketone (PEEK). In the experimental setup, a pair of excitation coils was wound around the housing of the miniaturized magnetometer probe. The excitation magnetic field was generated by these coils, driven by a power supply and modulated by the signal generator. The modulated excitation field was aligned in the same direction as the optical signal. The miniaturized magnetometer probe, with the excitation coils wound around it, was placed inside the triaxial Helmholtz coil and the magnetic shielding barrel to mitigate external magnetic field interference. The MEMS vapor cell was heated on both sides by nonmagnetic heating elements, while a nonmagnetic platinum resistor located at the bottom of the vapor cell continuously monitored the temperature in real time. The measured temperature values were fed back to the temperature controller, enabling closed-loop control to ensure the thermal stability of the vapor cell. The laser, emitted from the optical fiber, was collimated by a Grin Lens at the front end of the fiber and subsequently passed through a polarizer, a polarizing beam splitter (PBS), and a quarter-wave plate, converting the laser into circularly polarized light. The laser then traversed the MEMS vapor cell and reached the photodetector (PD). The PD converted the optical signal into an electrical current, which was amplified by the transimpedance amplifier and fed into the lock-in amplifier. The lock-in amplifier demodulated the output signal from the transimpedance amplifier based on the applied modulation, thereby extracting the desired magnetic field information.

**Figure 2:**
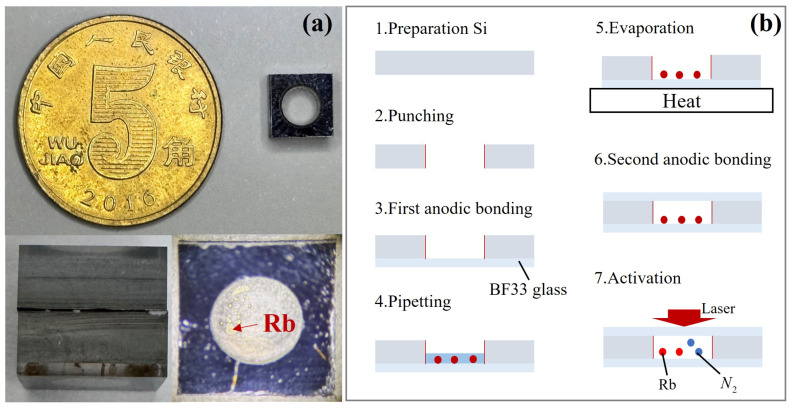
Physical Representation of the alkali–metal MEMS vapor cell (**a**) and the overall fabrication process (**b**).

The fabrication process for thick MEMS vapor cells was similar to those reported in the literature [22,23] and can be broadly divided into the following steps: wafer preparation, drilling, first anodic bonding, liquid dispensing, heating and evaporation, second anodic bonding, and alkali metal atom activation. In this study, double-sided polished silicon wafers and BF33 glass with a diameter of 4 mm and a thickness of 5 mm were used, as shown in Figure 2a. The overall fabrication process is illustrated in Figure 2b. The detailed steps of the process are described below. The fabrication process began with wafer preparation, where high-quality silicon wafers with appropriate thickness were selected as the substrate material for the MEMS vapor cell. The wafer thickness determined the depth of the vapor cell, while its surface quality directly affected bonding integrity and hermeticity. Thick silicon wafers offered superior mechanical strength and stability, facilitating the formation of precise vapor cell structures and enhancing the device’s durability and long-term stability. To ensure optimal performance in the subsequent processing steps, the wafer surface was cleaned and treated to remove particles and contaminants that could compromise fabrication quality. Drilling was a critical step in the fabrication process for creating channel structures within the silicon wafer. For thick MEMS vapor cells, which require deep and wide channels, drilling is more suitable than etching. This method enables the rapid formation of straight and smooth deep channels in thicker wafers, ensuring smooth channel edges and reduced sidewall roughness. These characteristics improve the hermeticity and dimensional accuracy of the channels. Moreover, the mechanical stress introduced by drilling is minimal, helping to maintain the structural integrity of the vapor cell. Anodic bonding is a process that tightly bonds silicon and glass through the application of electric fields and heat. In this study, the first anodic bonding step was performed between the drilled silicon wafer and BF33 glass to form the initial vapor cell structure. Before bonding, the silicon wafer and glass were subjected to RCA standard cleaning and drying. The cleaned components were then placed into the bonding equipment’s slots, heated to 350 °C, and subjected to a pressure of 4500 kN to ensure close contact between the silicon and glass. Once the pressure stabilized, the voltage was incrementally increased from 0 V to 1200 V in a stepwise manner. The bonding process was completed when the current decreased to near 0 V. This anodic bonding step ensured the preliminary sealing and structural stability of the vapor cell, effectively preventing external vapor and impurities from entering. Additionally, it provided a robust sealed structure, ensuring the vapor cell’s hermeticity. After cooling to room temperature, an aqueous solution of rubidium azide was introduced into the chamber, with a total rubidium azide fill amount of 12 µg. The vapor cell was then heated to evaporate the injected liquid material. By precisely controlling the temperature and duration of the process, the desired concentration and uniform distribution of vapor content within the vapor cell were ensured. Under vacuum conditions, the previous steps were repeated to perform the second anodic bonding, completing the triple-layer glass–silicon–glass bonding process. Once cooled to room temperature, the bonded wafer stack was segmented into individual MEMS vapor cells using a laser dicing machine, as shown in Figure 2a. Following packaging, the vapor cells were activated by high-energy laser irradiation. This activation process caused alkali metal rubidium atoms and inert vapor (nitrogen) within the cell to desorb and diffuse, creating an active vapor environment essential for operation.

To achieve high atomic density, MEMS vapor cells require heating to elevated temperatures, necessitating the use of high-temperature-resistant materials for the sensor housing to ensure stability and prevent deformation. Polyether ether ketone (PEEK) was selected as the housing material due to its high strength, rigidity, and exceptional thermal resistance. Countersunk screws made of the same PEEK material were used to secure the Grin lens, PBS, and MEMS vapor cell, ensuring structural integrity and thermal compatibility.

## 3. Results and Discussion

The free induction decay (FID)-based atomic magnetometer leverages the precession of atomic spins in an external magnetic field to achieve high-sensitivity magnetic field measurements [24,25]. Initially, an excitation magnetic field is applied along the optical axis to polarize the atomic spins. This polarization process aligns the spins with the excitation field, generating a macroscopic magnetic moment in the atomic vapor. When the excitation field is turned off, the atomic spins retain their orientation along the initial polarization direction. Upon applying a weak magnetic field in space, the atomic magnetic moment undergoes Larmor precession about the direction of the weak field, with the Larmor frequency determined by the strength of the external magnetic field and the gyromagnetic ratio of the atoms. The excitation magnetic field signal is amplitude-modulated (AM) using a pulse width modulation (PWM) signal. During the high-level phases of the PWM signal, the excitation magnetic field is active, whereas during the low-level phases, it is turned off. When the excitation magnetic field is deactivated, the polarized atoms sense the magnetic field applied in space and precess around its direction. The relationship between the Larmor frequency and the magnetic field strength is described by Equation (1).

(1) $$\gamma \cdot \omega_{L}=B$$

where *B* represents the magnetic field applied perpendicular to the optical axis, $\omega_{L}$ denotes the Larmor precession frequency of the atoms, and γ is the gyromagnetic ratio of rubidium-87, given as $4.67\;\mathrm{Hz}\cdot {\mathrm{nT}}^{-1}$.

**Figure 3:**
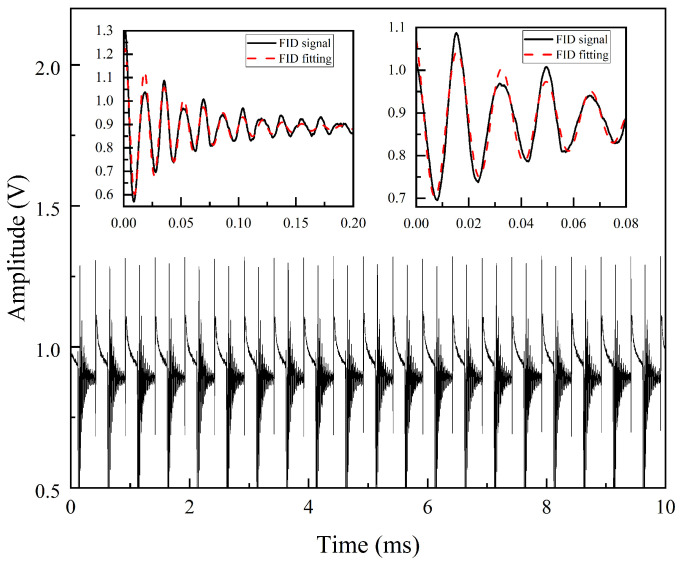
Free induction decay (FID) signal.

During the excitation signal’s active phase, the exciting field and the polarization effect of the laser lead to the coherence of the atomic spins. However, interactions among atoms and external perturbations gradually disrupt this coherence. As a result, the phase distribution of the atomic spins diffuses when the excitation field is turned off, and the polarization precession diminishes over time. This loss of coherence causes the magnetic moment signal to decay, forming the free induction decay (FID) signal. In the experiment, a PWM modulation signal with a peak-to-peak voltage of 4 V, a bias voltage of 2 V, a frequency of 2 kHz, and a duty cycle of 40% is applied. During each low-level period of the PWM signal, the FID signal is recorded and fitted. Consequently, the FID signal in the time domain also exhibits a periodicity of 2 kHz, as illustrated in Figure 3.

The FID signal fitting process involves multiple steps, including data preprocessing, signal segmentation, and parameter fitting. The primary objective is to extract physical parameters more accurately in a noisy environment, thereby enhancing the precision of signal analysis. First, the FID signal must be extracted from the raw data. To achieve this, a trigger signal is defined for segmentation, with threshold values adjusted for FID signals under different magnetic fields. The trigger point is identified by detecting when the raw signal drops below the set threshold, capturing the descending edge of the signal. The first panel in Figure 3 shows a complete FID signal cycle following the deactivation of the excitation magnetic field. In this instance, the vapor cell is heated to 105 °C, and the current applied to the *z*-axis coil is 100 mA. Initially, residual effects from the excitation pulse may influence the signal, causing transient effects such as amplitude and phase shifts, which deviate from the ideal exponential decay model in the first few cycles. In the later stages of the FID signal, the amplitude decays significantly, leading to large data fluctuation and inaccuracies in parameter estimation, such as the fitted frequency $\omega_{L}$ and transverse relaxation time $T_{2}$. To mitigate these effects, discarding the initial cycles can eliminate residual excitation interference, while removing the final cycles avoids low-SNR regions, ensuring a more accurate representation of the oscillatory decay process. For fitting, the high-SNR segment of the FID signal is selected by defining a high-level trigger window. The second panel in Figure 3 shows the data segment extracted from the FID signal in the first panel, which is used for fitting. The trigger signal threshold is set to 0.69 V, and the high-level trigger window is configured with a duration of 80 µs. After extracting the fitting data, Equation (2) is applied to fit the free induction decay signal following the termination of the excitation magnetic field. The fitted frequency is then used to calculate the magnetic field value, which is determined to be 10,924 nT, with a transverse relaxation time $T_{2}$ of approximately 70 µs. Compared to fitting an entire decay cycle, this approach yields more precise frequency estimates, resulting in more accurate magnetic field measurements. Finally, the fitted frequency is multiplied by the gyromagnetic ratio using Equation (1) to calculate the magnetic field, enabling external field detection.

(2) $$y=A_{1}\cdot e^{-t/T_{2}}\cdot \sin\left(2\pi \omega_{L}t+\phi \right)+A_{2}$$

where $A_{1}$ represents the amplitude; $T_{2}$ denotes the transverse relaxation time of the atoms, which determines the signal decay rate; and $\omega_{L}$ is the fitted Larmor precession frequency of the atoms, related to the strength of the external magnetic field. ϕ is the phase factor, and $A_{2}$ is the offset, accounting for the constant shift in the fitted signal.

**Figure 4:**
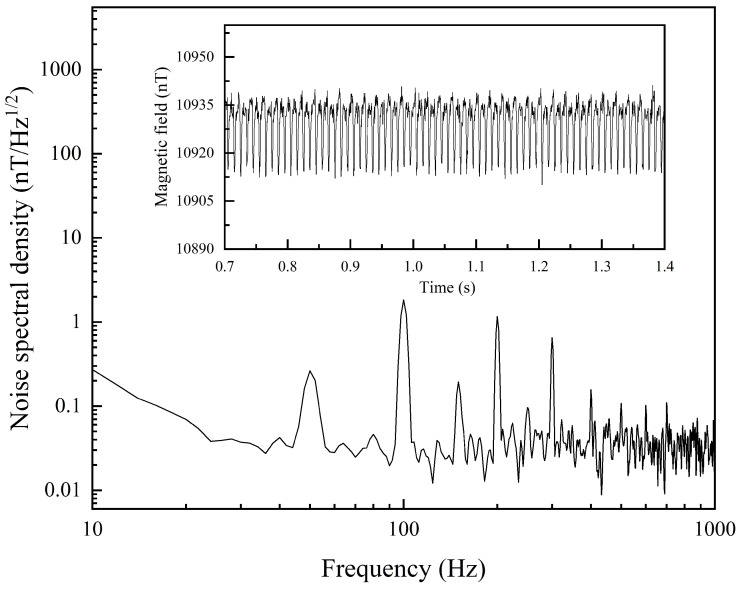
Sensitivity of scalar magnetic field detection.

In the experiment, a data acquisition card was used to capture the PD signal over a period of time. With a sampling rate of 10 MHz, the data acquisition card recorded the FID signals, and the middle segments of each cycle were extracted for fitting, yielding a series of time-domain magnetic field data, as shown in the inset in Figure 4. By performing power spectral density (PSD) analysis on the fitted magnetic field data, the scalar magnetic field sensitivity was determined. Our scalar magnetic field sensitivity reached 30 pT·Hz^−1/2^.

**Figure 5:**
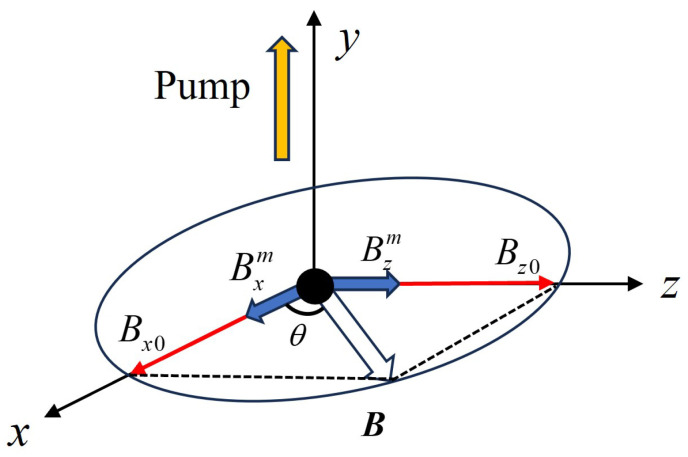
Schematic diagram of vector magnetic field detection.

FID-based atomic magnetometers are primarily utilized for scalar magnetic field detection, measuring the magnitude of the magnetic field rather than its direction. The operational principle relies on detecting the Larmor precession frequency of atomic spins in an external magnetic field to determine the field’s magnitude. As a result, the output signal is solely related to the magnetic field strength and does not contain directional information. By introducing modulation techniques into FID-based scalar atomic magnetometers, it becomes possible to decouple the magnetic field components step by step, enabling vector magnetic field detection. This advancement significantly broadens the application scope of FID-based atomic magnetometers, providing more comprehensive magnetic field information. This approach retains the high-sensitivity and low-noise characteristics of FID magnetometers while enabling the measurement of magnetic field direction, offering a compact and efficient solution for vector magnetic field measurements. Figure 5 illustrates our method for vector magnetic field detection. Building upon high-sensitivity scalar magnetic field detection, this study implemented two modulation fields along the *x* and *z* axes perpendicular to the optical axis. By demodulating the resulting signals, the vector magnetic field within the *x*–*z* plane can be measured, including both the magnitude and direction of the field. Assuming a vector magnetic field B exists in the *x*–*z* plane, its components along the *x* axis and *z* axis are represented as $B_{x}$ and $B_{z}$, respectively, as illustrated in Figure 5. When modulation fields are applied along the *x* and *z* axes, the magnetic field components $B_{x}$ and $B_{z}$ can be expressed using Equations (3) and (4).

(3) $$\begin{array}{cc} B_{x} & =B_{x0}+B_{x}^{m}\sin\left(2\pi \omega_{x}t\right) \end{array}$$

(4) $$\begin{array}{cc} B_{z} & =B_{z0}+B_{z}^{m}\sin\left(2\pi \omega_{z}t\right) \end{array}$$

where $B_{x}^{m}$ and $B_{z}^{m}$ represent the modulation amplitudes applied to the *x* and *z* axes, respectively, while $\omega_{x}$ and $\omega_{z}$ denote the modulation frequencies of the fields applied along these axes. With the introduction of modulation fields, the vector magnetic field B within the *x*–*z* plane can be expressed using Equation (5), and the square of the magnetic field can be represented by Equation (6).

(5) $$B=B_{x}+B_{z}$$

(6) $$\begin{array}{cc} B^{2} & ={\left(B_{x0}+B_{x}^{m}\sin\left(2\pi \omega_{x}t\right)\right)}^{2}+{\left(B_{z0}+B_{z}^{m}\sin\left(2\pi \omega_{z}t\right)\right)}^{2}\\ \; & =B_{x0}^{2}+B_{z0}^{2}+2B_{x0}B_{x}^{m}\sin\left(2\pi \omega_{x}t\right)+2B_{z0}B_{z}^{m}\sin\left(2\pi \omega_{z}t\right)\\ \; & \;+{\left(B_{x}^{m}\sin\left(2\pi \omega_{x}t\right)\right)}^{2}+{\left(B_{z}^{m}\sin\left(2\pi \omega_{z}t\right)\right)}^{2} \end{array}$$

By demodulating the square of the fitted magnetic field at frequencies $\omega_{x}$ and $\omega_{z}$, the magnetic field components $2B_{x0}B_{x}^{m}$ and $2B_{z0}B_{z}^{m}$ can be extracted. Subsequent filtering removes the high-frequency components, isolating the desired signal. When modulation fields of known amplitude and frequency are applied, the magnetic field components along the *x* and *z* axes in the *x*–*z* plane, $B_{x0}$ and $B_{z0}$, can be determined. Using these components, the magnitude of the vector magnetic field B and its angle θ relative to the *x* axis can be calculated using Equation (7). By applying modulation signals along two cross directions, vector magnetic field detection based on the FID atomic magnetometer is achieved, enabling the precise measurement of both the magnitude and direction of the field in the plane perpendicular to the optical axis.

(7) $$\left\{\begin{array}{c} B=\sqrt{B_{x0}^{2}+B_{z0}^{2}}\\ \theta =90^{\circ }-\arctan\left(\frac{B_{z0}}{B_{x0}}\right) \end{array}\right.$$

**Figure 6:**
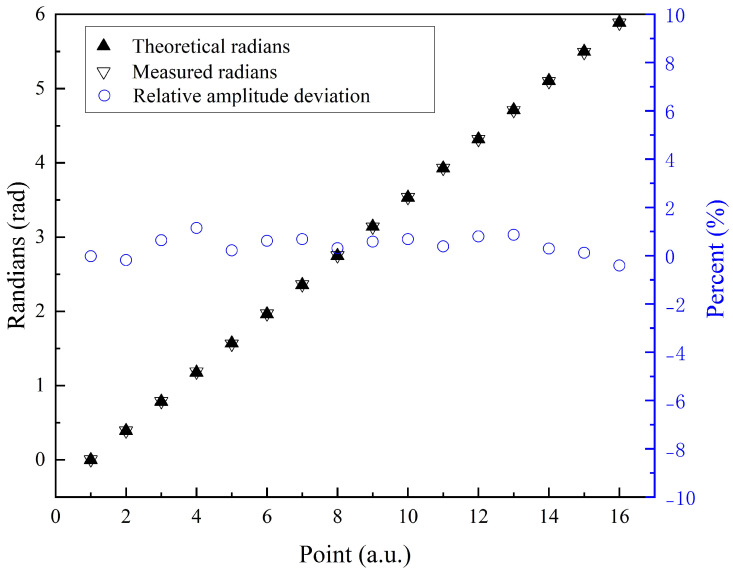
The result of the in-plane vector measurement.

**Figure 7:**
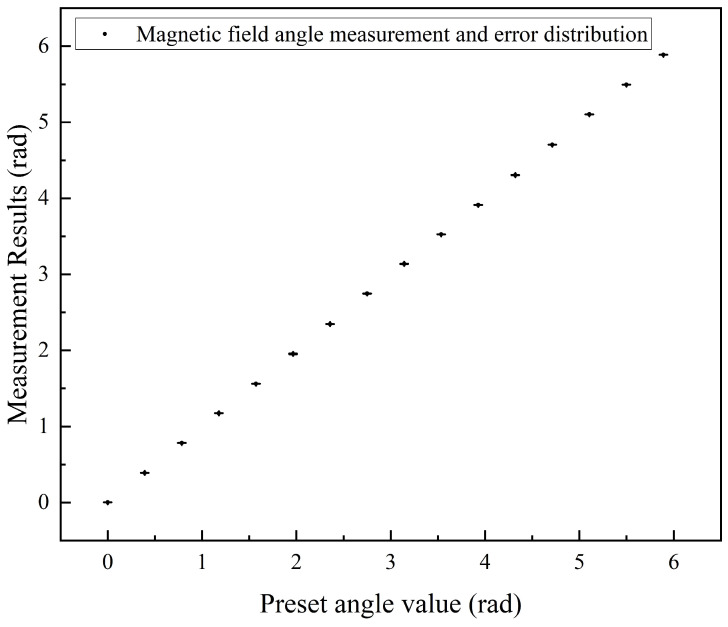
Magnetometer system performance statistics.

A series of magnetic fields with constant magnitudes and varying angles were applied within the plane perpendicular to the optical axis. For each magnetic field angle, the PD signal was recorded over a specific time period, and the FID signals for each cycle were extracted and fitted to determine the magnetic field values. The fitted data were then demodulated along the *x* and *z* axes to obtain the magnetic field components. Using Equation (6), the angle of the detected magnetic field was calculated. The deviations between the measured magnetic field angles relative to the *x* axis and the preset vector magnetic field angles, calculated based on the coil currents, are shown in Figure 6. For the magnetic field magnitude, Figure 6 also shows the percentage deviation in the measured field strength. The left vertical axis represents the angle in radians, while the right vertical axis indicates the percentage measurement error. The average angular measurement error for the in-plane vector magnetic field was 4.7 mrad. The field strength average measurement error percentage was 0.49%. These results confirm that by applying two modulation fields, the FID-based atomic magnetometer can be effectively configured as a vector magnetometer, enabling accurate detection of vector magnetic fields within the plane perpendicular to the optical axis. Building upon the vector magnetic field measurements shown in Figure 6, we conducted five sets of repeated measurements at each angle. For each angle, the average value of the five measurements was calculated, and the standard deviation of the measurement error was determined. Figure 7 presents the vector magnetic field measurement results across the full range of angles, with error bars representing the standard deviation at each angle. Based on the results, the average measurement error of the vector magnetic field across 16 angles was 5.6 mrad, with an average standard deviation was 0.83 mrad.

**Figure 8:**
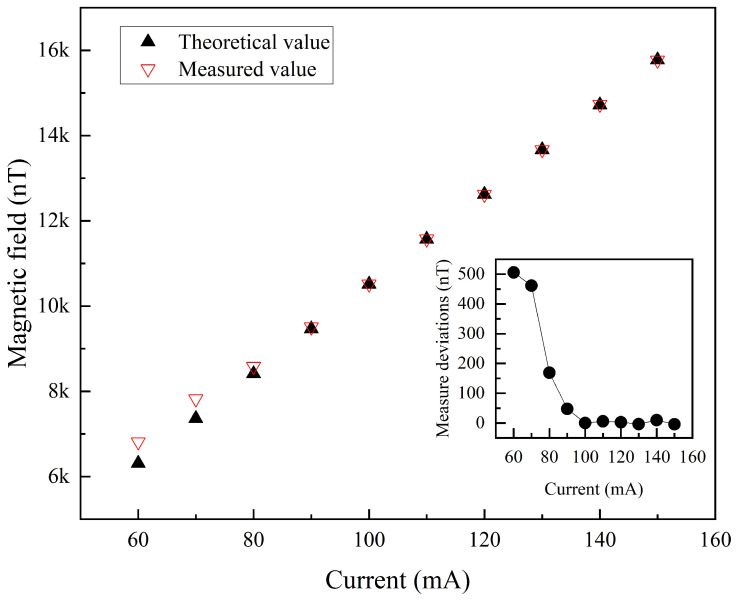
Measured and preset values of the test magnetic F = field.

The accuracy of magnetic field fitting is influenced by the strength of the applied field. For weaker fields, the Larmor precession frequency is lower. In this case, the FID signal exhibits fewer oscillation decay cycles within the limited time after the excitation field is turned off, potentially leading to suboptimal fitting accuracy. Conversely, for stronger fields, the higher Larmor precession frequency yields a higher SNR and a greater number of oscillation decay cycles within the same time frame. This improves the fitting accuracy, reducing the discrepancy between the fitted and preset magnetic field values. As shown in Figure 8, when the current of the applied test field increased from 60 mA to 150 mA, the fitted magnetic field values gradually converged with the preset values. Once the applied field current exceeded 100 mA, the error between the preset and measured values converged toward zero. The inset in Figure 8 illustrates the trend in the measurement deviations as the applied field current increased from 60 mA to 150 mA. For currents of 100 mA or higher, the deviation in the measured magnetic field remained within 10 nT, ensuring the measurement precision of the FID-based vector atomic magnetometer.

The magnetometer system proposed by B. Patton applies two orthogonal light-shift laser beams along the *y* and *z* axes, enabling high-sensitivity vector magnetic field measurements in two directions (*y* and *z*) by demodulating changes in the Larmor frequency. However, the system’s structure is relatively complex. The literature also notes that by introducing an additional light-shift laser along the *x* axis, the system can be extended to achieve three-axis vector magnetic field detection. Similar to the approach presented in this paper, two modulations are employed to enable two-dimensional vector magnetic field detection. By extending the modulation scheme, three-axis vector magnetic field detection can also be realized. In contrast, the magnetometer proposed by Xin Meng, which utilizes machine-learning-assisted nonlinear magneto-optical rotation (NMOR), and the system introduced by Zhang JiaLong, based on coordinate system rotation in a SERF atomic magnetometer, are both capable of three-axis vector magnetic field detection. However, the NMOR-based magnetometer leveraging machine learning is limited by the linewidth of the resonance, while the SERF atomic magnetometer based on coordinate system rotation is constrained by interaxis coupling effects, which restrict the system response. And, our miniaturized magnetometer still encounters several challenges. At present, the buffer gas pressure in the MEMS vapor cell is limited to 1 atm. Under these conditions, rubidium atoms undergo more frequent collisions with the cell walls, resulting in enhanced wall-induced relaxation and a reduction in spin relaxation time. This leads to a rapid loss of atomic spin coherence, further degrading the sensitivity of magnetic field detection.

**Figure 9:**
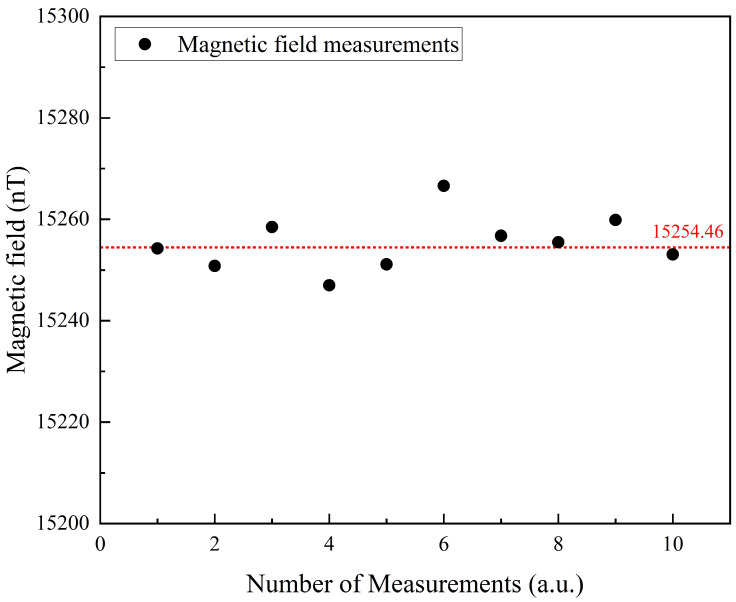
Magnetometer system reliability analysis.

To evaluate the reliability of the magnetometer system, a target magnetic field of 15,254.46 nT was applied. The laser was activated, the vapor cell was heated to the predetermined temperature, and an excitation magnetic field signal was applied. The PD signal was acquired, and the FID signals were fitted to determine the measured magnetic field values, as shown in Figure 9. Following the fitting process, the vapor cell heating, excitation magnetic field, laser, and target magnetic field were deactivated. After the vapor cell cooled to room temperature, the heating, laser, excitation magnetic field, and target magnetic field were reactivated, and the FID signals were fitted again. This procedure was repeated 10 times to verify the reliability of the magnetometer system. The average value of the target magnetic field over the 10 measurements was 15,254.25 nT, with an average measurement deviation of 4.72 nT. These results demonstrate the stability and consistency of the magnetometer system. The primary source of measurement error is attributed to deviations in the FID signal fitting process, highlighting the influence of fitting accuracy on overall measurement precision.

## 4. Conclusions

In summary, this study presents a free induction decay (FID)-based vector atomic magnetometer utilizing a microfabricated vapor cell. The 5 mm thick MEMS vapor cell was fabricated using mechanical drilling and anodic bonding techniques, and the magnetometer was miniaturized with customized optical components. Building on scalar magnetic field detection, the system incorporates cross-modulation techniques to enable the simultaneous measurement of magnetic field strength and direction. The proposed method demonstrated a total magnetic field sensitivity of 30 pT·Hz^−1/2^ @11 µT under magnetically shielded conditions. Highlighting its capability for accurate vector magnetic field detection, the miniaturized atomic magnetometer achieved an average angular deviation of 4.7 mrad @7.6 µT for vector magnetic field measurements. The system exhibited exceptional sensitivity and directional resolution in weak magnetic field environments. Statistical analysis of the vector magnetic field measurements revealed an average measurement error of 5.6 mrad with an average standard deviation of 0.83 mrad. To evaluate the reliability of the miniaturized magnetometer system, the system was periodically activated and deactivated, and the same target magnetic field was measured. After ten repeated measurements, the average value of the measured magnetic field was 15,254.25 nT, with an average measurement error of 4.72 nT. In future work, we plan to fill the MEMS vapor cell with buffer gas at low temperatures, resulting in the buffer gas pressure significantly exceeding atmospheric pressure at room temperature. This approach is expected to reduce the collisions between rubidium atoms and the cell walls, thereby increasing the atomic spin relaxation time. As a result, the amplitude decay of the FID signal will slow, extending the signal duration. This improvement is anticipated to enhance the accuracy of FID signal fitting and further improve the sensitivity of the miniaturized magnetometer system. Additionally, further optimization of FID signal fitting algorithms, vapor cell temperature, and laser power will be explored to further enhance the sensitivity of the system. We also aim to integrate new modulation schemes to enable three-axis vector magnetic field detection. These advancements are intended to expand the magnetometer’s applications in geophysical exploration, aerospace navigation, and biomedical research.

## Data Availability

The original contributions presented in this study are included in this article; further inquiries can be directed to the corresponding author.

## Abbreviations

The following abbreviations are used in this manuscript:

FID	Free induction decay
MEG	Magneto-encephalography
RMS	Root mean square
SERF	Spin-exchange relaxation-free
PD	Photodetector
AM	Amplitude-modulated
PWM	Pulse width modulation
PSD	Power spectral density
PEEK	Polyether ether ketone
